# Comparison of Biochemical Parameters between Mouse Model and Human after Paraquat Poisoning

**DOI:** 10.1155/2022/1254824

**Published:** 2022-01-28

**Authors:** Jielun Yu, Lichun Zhang, Xiaoshuang Li, Kaixuan Lv, Shiyu Sun, Weihua Wu, Lifeng Ping, Guifang Guo, Wei Tan, Shoudong Guo, Kezhou Wang, Aihua Zhao, Nana Yang

**Affiliations:** ^1^School of Life Science and Technology, Weifang Medical University, Weifang, Shandong, China; ^2^Medical Laboratory Animal Center, Weifang Medical University, Weifang, Shandong, China; ^3^Weifang Key Laboratory of Animal Model Research on Cardiovascular and Cerebrovascular Diseases, Weifang, Shandong, China; ^4^School of Clinical Medicine, Weifang Medical University, Weifang, Shandong, China; ^5^Department of General Practice, The Second Affiliated Hospital of Shandong First Medical University, Taian, Shandong, China; ^6^Weifang People's Hospital, Weifang, Shandong, China; ^7^School of Pharmacy, Weifang Medical University, Weifang, Shandong, China; ^8^School of Laboratory Animal, Shandong First Medical University & Shandong Academy of Medical Sciences, Jinan, China

## Abstract

**Background:**

This study was designed to investigate differences in biochemical parameters between mouse and humans after paraquat (PQ) poisoning and develop a suitable animal model for studying organ damage after PQ poisoning. The prognostic factors of PQ-poisoned patients were further analyzed.

**Methods:**

Thirty C57BL/6J mice were randomly divided into five groups (control, sham, and 3 PQ doses), and the mouse model was established by intragastric administration of PQ. Physiological indexes such as the body weight, mental state, and mortality rate were observed. Biochemical parameters were analyzed 24 h after PQ poisoning. We also performed a retrospective analysis of clinical data from 29 patients with PQ poisoning admitted to the Emergency Department of the Affiliated Hospital of Taishan Medical College between April 2016 and February 2018. Biochemical parameters were compared between the mouse model and patients with PQ poisoning.

**Results:**

In the PQ poisoning mouse model, the lethal dose group PQ360 showed remarkable increases in serum levels of potassium (K^+^), carbon dioxide (CO_2_), alanine aminotransferase (ALT), and aspartate aminotransferase (AST) compared with the nonlethal dose PQ100 and PQ200 groups. The biochemical results of the patients showed that K^+^ and Cl^−^ levels were significantly reduced in the death group compared to the survival group. Levels of ALT, AST, blood urea nitrogen (BUN), and amylase were higher, and the neutrophil-to-lymphocyte ratio (NLR) was increased in the death group compared with the survival group.

**Conclusions:**

The combination of age, PQ dosage, K^+^, Cl^−^, BUN, ALT, AST, amylase, and NLR can be used to more accurately predict the outcome of patients with PQ poisoning. C57 mice are an appropriate animal model to study liver and kidney functions following PQ exposure.

## 1. Introduction

Paraquat (1, 1′-dimethyl-4, 4′-bipyridinium dichloride, PQ), also called Yisaoguang (in Chinese Pinyin) and Gramoxone, is a highly effective, nonselective, contact herbicide used widely worldwide. It can be quickly absorbed by the green tissues of plants and quickly withers the foliage. PQ is rapidly deactivated once combined with soil, so it does not affect the roots or perennial rhizomes of plants. This herbicide is extremely poisonous to humans and animals [1–3]. Intentional or unintentional PQ ingestion has an extremely high mortality rate [3]. The oral lethal dose of PQ for adults is 2-6 g, corresponding to 15-20 ml of the commonly used 20% aqueous PQ solution [4]. There is no effective antidote for PQ poisoning. Acute intoxication following PQ ingestion leads to multiple organ damage, affecting the lungs, liver, kidneys, and heart [5], and there is a high mortality rate due to the lack of effective therapeutic strategies. The precise mechanism of PQ intoxication remains elusive, which has hampered treatment development. A variety of animal species have been used as research models for PQ toxicology studies [2, 6]. Mice are the most widely used, so clarifying the differences in biochemical parameters between humans and mice is important for understanding PQ toxicological molecular mechanisms.

A number of predictors for prognostic outcome have been used in patients with PQ poisoning, including plasma PQ concentration [7], arterial lactate level and lactate metabolic clearance [8], the Acute Physiologic Assessment and Chronic Health Evaluation II (APACHEII) score [9], the Modified Simplified Acute Physiology Score II (MSAPS II) [10], the Sequential Organ Failure Assessment Score (SOFA), and the Severity Index of PQ Poisoning Score (SIPP). Unfortunately, many of these assessments are not readily available in smaller or rural hospitals, and they have poor sensitivity and specificity in some patients. At present, hospitals of all levels mainly provide symptomatic treatment for PQ poisoning, but there is a lack of unified standards. Treatment may be excessive for patients with mild symptoms but ineffective for severe patients [11].

This study is aimed at identifying markers for early judgment of PQ poisoning severity to improve diagnosis by conducting a retrospective analysis on clinical data from 29 patients who were diagnosed with oral PQ poisoning. We also evaluated the suitability of an animal model for studying organ damage after PQ poisoning by comparing biochemical parameters between animal model and humans. The results of such studies will be valuable for elucidating the mechanism of PQ poisoning.

## 2. Materials and Methods

### 2.1. Experimental Animals and Chemicals

Thirty adult male C57BL/6J mice (22-25 g) were obtained from the Medical Experimental Animal Center of Weifang Medical College. The animal house temperature was maintained at 22 ± 2°C with a 12 h light/dark cycle. All animals were acclimatized 1 week prior to experiment and had free access to food and water. The studies were performed in strict accordance with the Guide for the Care and Use of Laboratory Animals published by the Weifang Medical University. The protocol was approved by the Committee on the Ethics of Animal Experiments of Weifang Medical University (Ethical Code: 2020SDL154). PQ was purchased from Aladdin (Shanghai Aladdin Biochemical Technology Co., Ltd., China).

### 2.2. Experimental Protocol and Groups

C57BL/6J mice were divided randomly to five groups (*n* = 6 each): control group, mice did not receive any substance; sham group, mice were given physiological saline according to their body weight; experimental group 1 (PQ100), intragastric (i.g.) administration of PQ 100 mg/kg; experimental group 2 (PQ200), PQ 200 mg/kg i.g.; and experimental group 3 (PQ360), PQ 360 mg/kg i.g (Supplementary Figure 1).

### 2.3. Animal Weight and Mental State Analysis

After treatment, the mice were maintained for 1 month. Mouse body weight was measured daily with a standard laboratory scales. The general state of animals was observed and recorded, including behavior, appetite, breathing, activity stimulus-response, and hair condition. The mortality rates were also calculated.

### 2.4. Biochemical Parameter Analysis

At 24 hours after PQ exposure, blood samples were collected and placed at room temperature for 2 hours. Blood serum was collected after centrifugation (15 min, 3000 rpm) and stored at -20°C until they were assayed. Various biochemical parameters in serum were measured using an automatic biochemical analyzer (AU 640 Medical System, Olympus, Japan) according to the manufacturer's protocols. The biochemical parameters tested include alanine aminotransferase (ALT), aspartate aminotransferase (AST), alkaline phosphatase (ALP), total protein (TP), albumin (Alb), bilirubin, malondialdehyde (MDA), total organic carbon (TOC), and total cholesterol (TC). The average of three replicates was recorded for each sample. The damage caused by different concentrations of PQ was determined based on biochemical parameter changes.

### 2.5. Statement of Ethics and Clinical Data

This retrospective study was conducted in accordance with the Declaration of Helsinki and was approved by the Medical Ethic Committee of Taishan Medical University and the associated Institutional Review Board. The study was a retrospective review of the existing data, so informed consent was not required. Written informed consent acknowledging risks related to PQ poisoning and treatments were provided by all patients when they were first admitted to the hospital. Patient records and information were used anonymously.

In this study, we retrospectively selected 29 cases diagnosed with PQ poisoning by the Affiliated Hospital of Taishan Medical University between April 2016 and February 2018. There were 15 males and 14 females aged between 14 and 65, with a mean age of 45.52 years old. Volumes of PQ ingested ranged between 5 and 200 ml. All patients were gastrointestinally intoxicated and diagnosed in accordance with the Expert Consensus on Diagnosis and Treatment of Acute PQ Poisoning (2013) by the Chinese College of Emergency Physicians (CCEP). The specific diagnostic criteria were (1) history of PQ ingestion; (2) clinical manifestations: cough, chest distress, and expiratory dyspnea for patients with pulmonary injury, and hematuria and oliguria for patients with renal injury; (3) laboratory assays: PQ detected in blood and urine, hypoxemia and metabolic acidosis detected in blood gas analysis, increases of ALT, AST, bilirubin, creatinine (Cr), and blood urea nitrogen (BUN) on liver and kidney function tests.

### 2.6. Treatment Protocol

All patients were given the same treatments: poison elimination (gastric lavage, medicinal charcoal adsorption, mannitol for catharsis, and blood purification), antioxidant treatment (high-dose vitamin C and reduced glutathione), immunosuppressants (glucocorticoids, etc.), and routine anti-inflammatory therapy to protect important organs.

### 2.7. Inclusion and Exclusion Criteria

Inclusion criteria: (1) patients with acute PQ poisoning through oral intake confirmed on admission, (2) aged ≥ 14 and ≤65, and (3) complete data and clear prognostic outcomes with availability for a follow-up visit 6 months following poisoning.

Exclusion criteria: (1) history of ingesting other poisons; (2) history of severe diseases in organs such as the heart, liver, and kidneys that significantly influence the outcomes of PQ poisoning; (3) had received treatment in other hospitals before admission or halted treatment after admission; (4) decline to participate in the study; (5) pregnant or lactating; or (6) comorbid cancer.

### 2.8. Data Collection

Two physicians independently collected data on patients with PQ poisoning within 1 h of admission, including urinary PQ concentration, complete blood counts, C-reactive protein (CRP), procalcitonin, liver and kidney function, electrolyte levels, blood glucose, blood coagulation, and myocardial enzymogram. We compared these biochemical parameters with those measured in mice.

### 2.9. Statistical Analysis

GraphPad Prism 8.0 (GraphPad Inc., San Diego, CA, USA) and SPSS 16.0 (SPSS Inc., Chicago, IL, USA) software packages were used to conduct statistical analyses. One- and two-way analyses of variance (ANOVAs) were used to compare results among multiple groups. The general information and laboratory data (urinary PQ concentration, complete blood counts, liver and kidney function, blood coagulation, and electrolyte) of patients were analyzed with *t*-tests if they were normally distributed and with nonparametric tests if nonnormally distributed. *P* ≤ 0.05 was considered statistically significant for all analyses.

## 3. Result

### 3.1. Physiological Indicator Assays

The general state of the control and sham groups showed normal breathing, activity, and behavior; good appetite; and no weight loss. Within 24 hours of PQ exposure, mice in the experimental groups showed abnormalities that mainly manifested as slow movement [12], unstable gait, and increased eye and nose discharge. The PQ360 group showed the most obvious changes including hair loss, slow responses, lethargy, reduced activity, less food consumption, and decreases in weight and body temperature.

**Figure 1:**
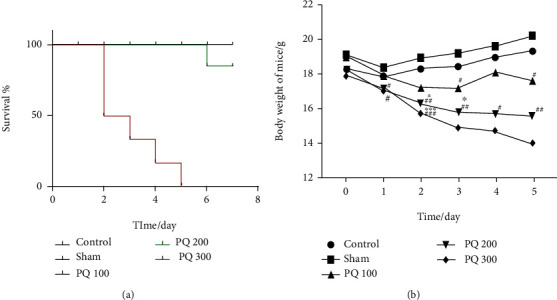
Effect of PQ on mouse body weight and survival rate. (a) Survival time was recorded over 7 days. (b) Body weight change was monitored for 5 days. PQ intoxication caused marked weight loss. Statistical analyses were performed using two-way ANOVA, and individual group differences were measured using Tukey's multiple comparisons tests. ^∗^*P* < 0.05, ^∗∗^*P* < 0.01, ^∗∗∗^*P* < 0.001 versus control; ^#^*P* < 0.05, ^##^*P* < 0.01, ^###^*P* < 0.001 versus sham.

The survival curves in Figure 1(a) show that the mortality rate of the PQ200 group 5 days after exposure was 16.7%. The mortality rate in the PQ360 group reached 50% on day 2 and was 100% at 5 days. After the third day, the number of surviving animals in the PQ360 group was less than three, so weight loss could not be statistically analyzed. Therefore, we only calculated body weight change in PQ360 group in the first two days after treatment.

In the first 5 days after PQ treatment, the weight of all experimental group mice was decreased (Figure 1(b)). On day 1, the body weight of mice in the PQ200 and PQ360 groups decreased significantly compared with the sham group. The weights of the PQ200 and PQ360 groups were significantly lower than the control and sham groups on day 2, but there was no difference in weight between the experimental groups. On day 3 after PQ administration, mice in the PQ200 group showed a significant decline compared with the sham and control groups. The PQ100 group weight was obviously lower than the sham group and showed a tendency to increase at day 4. Compared with the sham group, the mean weight of the PQ200 group was significantly reduced. By day 5, the body weight of mice in the PQ100 and PQ200 groups had decreased significantly. Five days later, the PQ200 and PQ100 group mice showed plateaued weight gain as compared to the control group (Supplementary Figure 2).

### 3.2. Biochemical Function Assays

**Figure 2:**
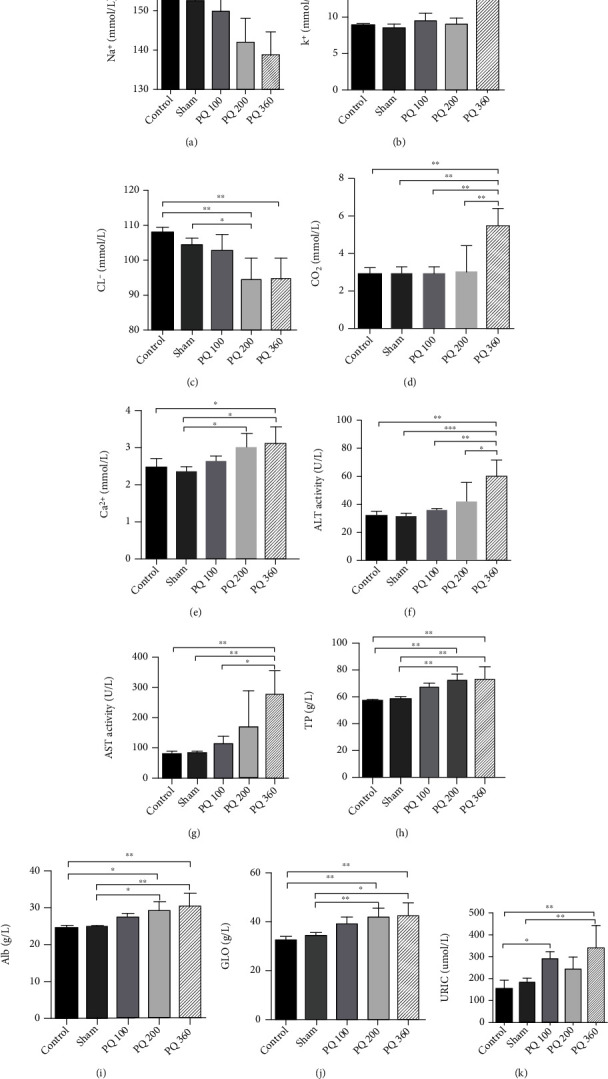
Biochemical function assays in the PQ poisoning mouse model. (a) Na^+^; (b) K^+^; (c) Cl^−^; (d) CO_2_; (e) Ca^2+^; (f) ALT; (g) AST; (h) TP; (i) Alb; (g) GLO; (k) uric acid. ^∗^*P* < 0.05, ^∗∗^*P* < 0.01, ^∗∗∗^*P* < 0.001.

PQ administration in the PQ200 group (nonlethal dose) caused a remarkable reduction (*P* < 0.05) in serum Na+ compared with the control, sham, and PQ100 groups (Figure 2). The Cl+ level of the PQ200 group was significantly lower than in the control and sham groups (*P* < 0.05). Serum Ca2+ in the PQ200 group was significantly higher compared with the sham group (*P* < 0.05). Compared with the control and sham groups, there were significant increases in levels of TP, Alb, and GLO in the PQ200 group. Uric acid in the PQ100 group was significantly elevated compared to the control group (*P* < 0.05). No significant changes in the experimental groups were observed for other indicators such as ALP, serum sialic acid, total bilirubin, direct bilirubin, serum indirect bilirubin, total bile acid, BUN, Cr, or *β*2-microglobulin (Supplementary Figure 3).

### 3.3. General Patient Information

According to the outcome, the 29 cases were divided into death (*n* = 10, 33.48%) and survival (*n* = 19, 65.52%) groups. Patients' conditions and laboratory results are shown in Table 1.

**Figure 3:**
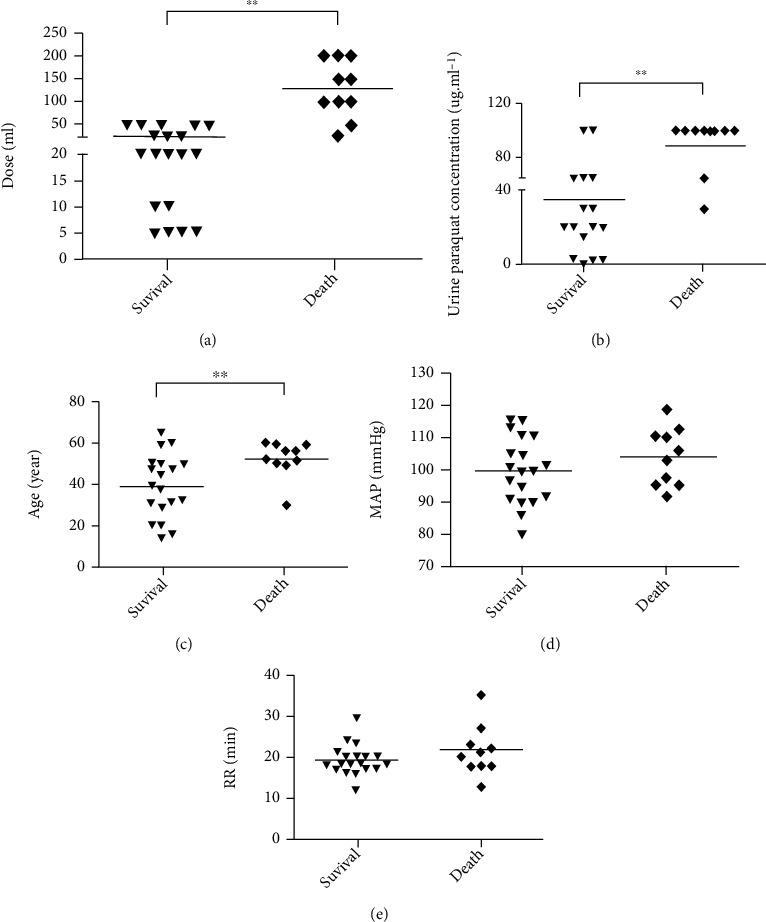
General condition of patients with PQ poisoning on admission. (a) Ingested volume; (b) urinary PQ concentration; (c) age; (d) mean arterial blood pressure; (e) respiration frequency. ^∗^*P* < 0.05, ^∗∗^*P* < 0.01.

Assessment of the general conditions of patients on admission revealed significant differences in volumes of PQ ingested, urinary PQ concentrations, and age between groups (Figure 3(a)). Patients who ingested a lower volume of PQ had a higher survival rate (25.26 ± 17.28 ml vs. 128.00 ± 61.97 ml, *P* < 0.01). Patients with lower urinary PQ concentrations also had a higher survival rate (Figure 3(b)) (*P* < 0.01). The two groups differed significantly in age (*P* < 0.01, Figure 3(c)), with younger patients more likely to be in the survival group. Figures 3(d) and 3(e) show that there was no significant difference in mean arterial blood pressure and respiration frequency between the two groups, respectively.

**Figure 4:**
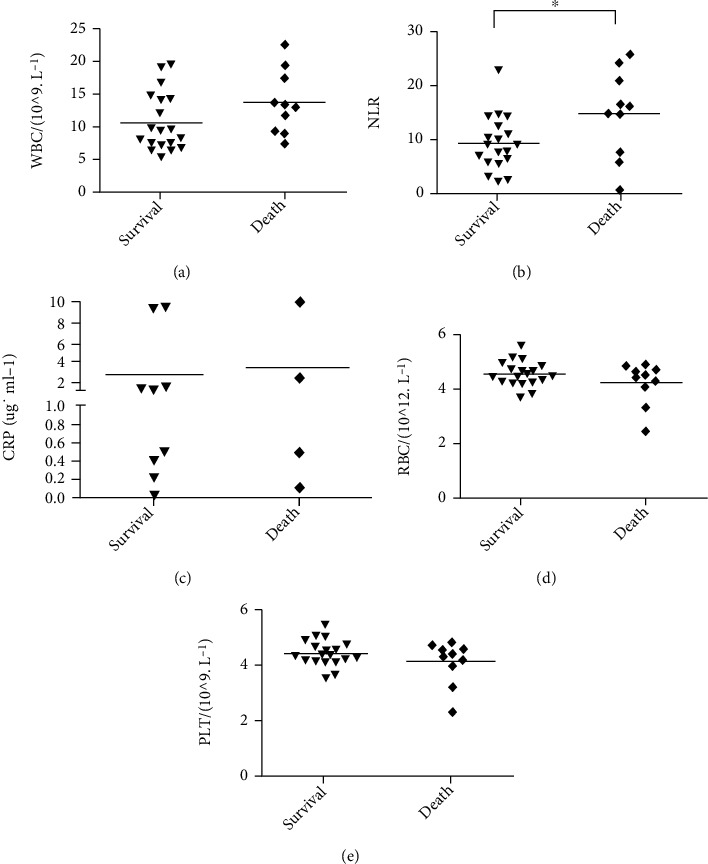
Complete blood count data of patients with PQ poisoning. (a) WBC: white blood cell; (b) NLR: neutrophil-to-lymphocyte ratio; (c) CRP: C-reactive protein; (d) RBC: red blood cell; (e) PLT: platelet. ^∗^*P* < 0.05, ^∗∗^*P* < 0.01.

Blood routine test results revealed that CRP levels and counts for white blood cells, red blood cells, and platelets were not significantly different between groups (Figure 4). The NLR was significantly different between the survival and death groups (9.46 ± 5.05 vs. 15.04 ± 8.14, respectively; *P* = 0.030; Figure 4(b)).

### 3.4. Comparing Biochemical Parameters between Mice and Humans

In the PQ poisoning mouse model groups, the comparison of the lethal dose group (PQ360) with the nonlethal dose groups (PQ100 and PQ200) showed that the K^+^, CO_2_, and ALT levels in the lethal dose group were substantially higher than for PQ100 and PQ200. There was a significant increase in AST in the lethal dose group compared to the PQ100 group (Figure 2(g)).

**Figure 5:**
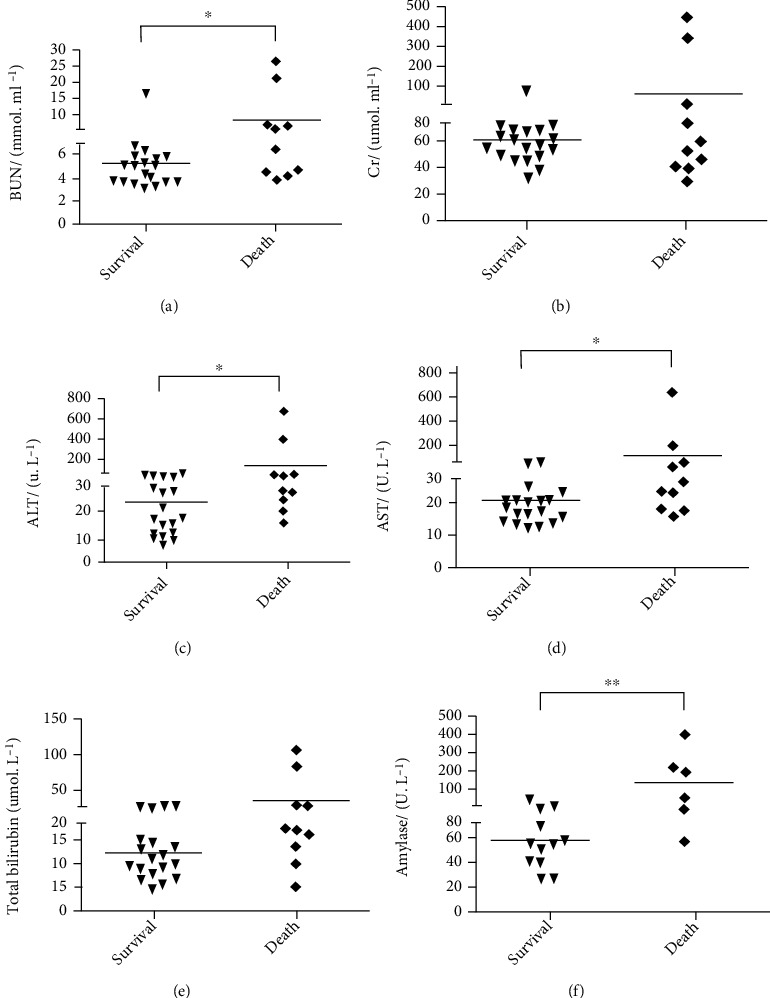
Liver function, kidney function, and serum amylase levels of patients with PQ poisoning. (a) BUN: blood urea nitrogen; (b) Cr: creatinine; (c) ALT: alanine aminotransferase; (d) AST: aspartate aminotransferase; (e) TBIL: total bilirubin; (f) amylase. ^∗^*P* < 0.05, ^∗∗^*P* < 0.01.

**Figure 6:**
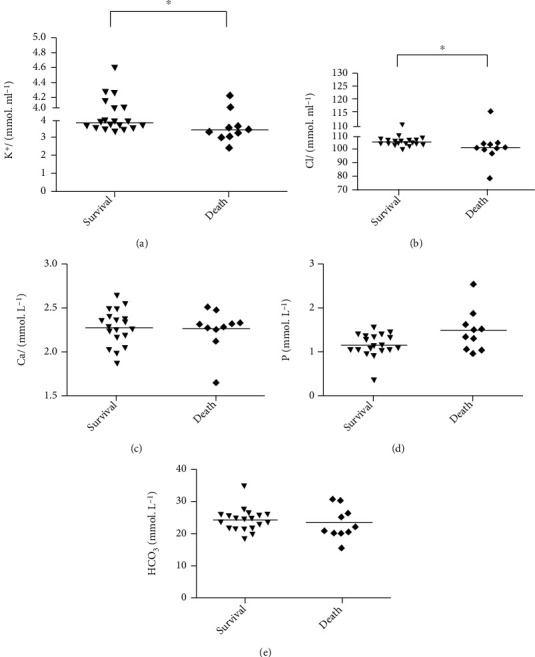
Serum electrolyte assays of patients with PQ poisoning. (a) K^+^: potassium; (b) Cl^−^: chlorine; (c) Ca^+^: calcium; (d) P: phosphorus; (e) HCO_3_^−^: bicarbonate. ^∗^*P* < 0.05, ^∗∗^*P* < 0.01.

**Figure 7:**
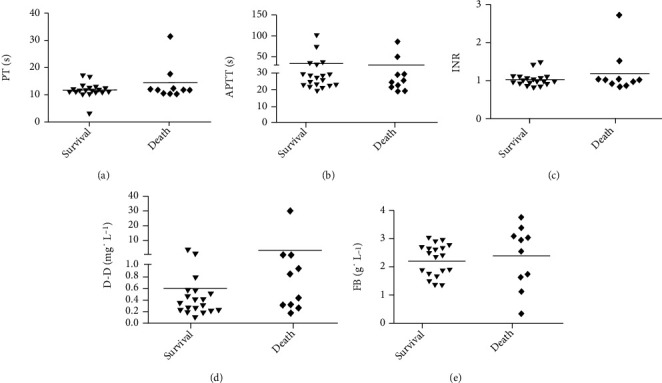
D-Dimer and coagulation assays of patients with PQ poisoning. (a) PT: prothrombin time; (b) APPT: activated partial prothrombin time; (c) INR: international normalized ratio; (d) D-Dimer concentration; (e) FIB: fibrinogen. ^∗^*P* < 0.05, ^∗∗^*P* < 0.01.

Patients' biochemical results (Figure 5) revealed that BUN, ALT, AST, and amylase levels differed significantly between the death and survival groups. The BUN values were 9.69 ± 7.96 mmol · ml^−1^ and 5.29 ± 3.05 mmol · ml^−1^, respectively (Figure 5(a)). The ALT of the death group was 137.30 ± 233.20 U · L − 1, which was significantly higher than 23.74 ± 15.03 U · L^−1^ in the survival group (*P* < 0.05, Figure 5(c)). The AST values of the survival and death groups were 22.05 ± 8.49 U · L^−1^ and 107.50 ± 201.41 U · L^−1^, respectively (*P* < 0.05, Figure 5(d)).  Figure 5(f) shows that serum amylase was significantly higher in the death group (211.33 ± 141.42 U · L^−1^ vs. 66.00 ± 29.32 U · L^−1^, *P* < 0.01).  Figure 5(b) shows that the difference in Cr levels between groups was not statistically significant, suggesting that Cr level is not associated with the survival of patients with PQ poisoning. Patient electrolyte data are shown in Figure 6. The mean serum K^+^ levels were 3.85 ± 0.30 mmol · ml^−1^ for the survival group, which was significantly higher than 3.45 ± 0.50 mmol · ml^−1^ in the death group (*P* < 0.05, Figure 6(a)). The serum concentrations of Cl^−^ (Figure 6(b)) for the death and survival groups were 101.30 ± 9.20 mmol · ml^−1^ and 105.58 ± 2.73 mmol · ml^−1^, respectively (*P* < 0.05). No significant differences were found between the two groups for serum concentrations of Ca^2+^, P, and HCO_3_^−^. There were also no remarkable differences in prothrombin time, activated partial prothrombin time, international normalized ratio, D-dimer concentration, or fibrinogen levels between the survival and death groups (Figure 7).

### 3.5. ROC Curve Analyses

**Figure 8:**
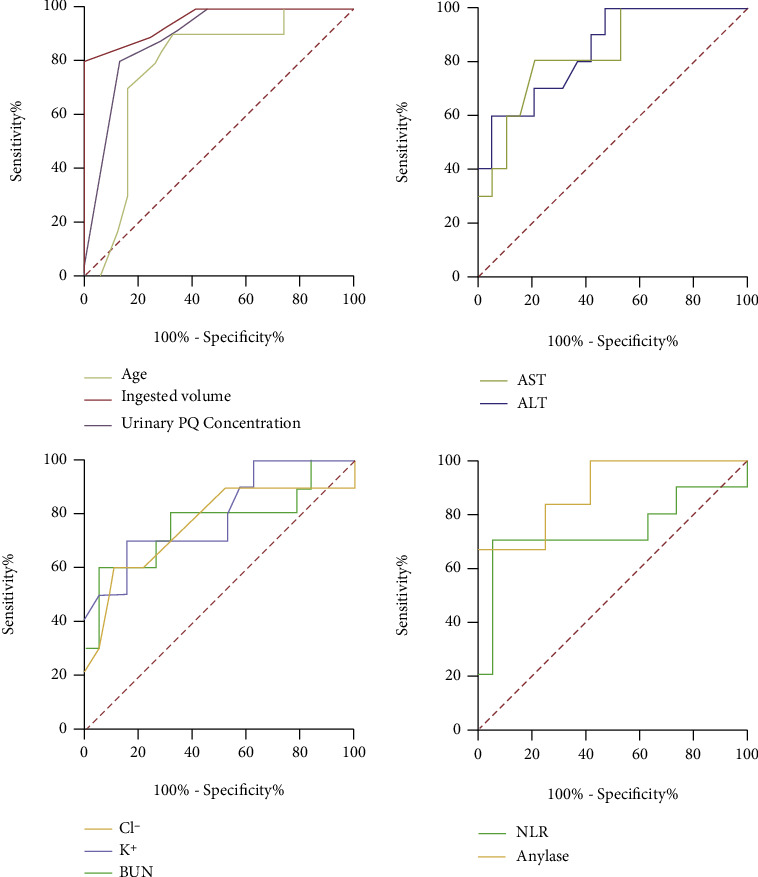
Receiver operating characteristic curves of multiple indicators for predicting mortality. ALT: alanine aminotransferase; AST: aspartate aminotransferase; BUN: blood urea nitrogen; Cl-: serum chlorine; K+: serum potassium; NLR: neutrophil-to-lymphocyte ratio.

ROC curve analysis was performed to evaluate the predictive ability of various indicators.  Table 2 shows that the areas under the ROC curve (AUCs) were 0.953 for ingested volume (95% confidence interval [CI]: 0.875-1.00, *P* < 0.0001), 0.889 for amylase (95% CI: 0.726-1.00, *P* = 0.0087), 0.883 for urinary PQ concentration (95% CI: 0.749-1.00, *P* = 0.0014), 0.845 for ALT (95% CI: 0.700-0.990, *P* = 0.0027), 0.832 for AST (95% CI: 0.677-0.986, *P* = 0.0038), 0.797 for BUN (95% CI: 0.619-0.976, *P* = 0.0095), 0.779 for age (95% CI: 0.597-0.961, *P* = 0.015), 0.763 for Cl^−^ (95% CI: 0.558-0.969, *P* = 0.0218), 0.761 for K^+^ (95% CI: 0.553-0.968, *P* = 0.0231), and 0.737 for NLR (95% CI: 0.504-0.970, *P* = 0.0389) (Figure 8). Except for NLR, all other indices had AUCs > 0.75, suggesting low predictive ability of NLR for PQ poisoning.

## 4. Discussion

PQ induces oxidative damage and cell death by generating ROS including hydrogen peroxide, superoxide anion, and hydroxyl radicals. Oral PQ ingestion impairs the lung, liver, kidneys, and neural tissues and eventually leads to death due to multiple organ failure [13]. Due to the lack of specific antidotes and chelating agents, an oral dose of 20 mg/kg can be lethal [4]. Animal modeling has played an essential role in exploring the underlying mechanism of PQ poisoning. One early study reported similar toxicity and symptoms of humans and animals suffering from PQ poisoning [14], but they did not describe specific indicators. Ensuring that animal models are relevant will improve investigation of the pathological mechanism(s) of PQ poisoning. Mice are commonly used to study the toxicological mechanism of PQ poisoning, so it is important to outline the similarities and differences of the toxicity mechanisms between mice and humans.

In this study, mice received i.g. PQ to simulate the clinical process of oral poisoning and digestive tract absorption. Liver function assays in patients and mice revealed significant differences in ALT and AST (*P* < 0.05). Compared with the PQ200 and PQ100 groups, both ALT and AST were significantly higher in the PQ360 group (lethal dose). Clinical statistical analysis showed that patients with higher ALT and AST concentrations were more likely to have a poor prognosis [15]. ROC curve analysis confirmed that ALT and AST were independent risk factors for mortality, which is consistent with findings reported by Bismuth et al. [16] and Kang et al. [17] that patients with higher ALT and AST concentrations had poorer outcomes. The liver is one of the main metabolizing organs, and PQ poisoning can damage it to varying degrees. Oxidative damage to hepatic vascular components occurs concomitantly with changes in hepatocyte cell membrane permeability after PQ poisoning. Traditionally, serum liver-specific enzyme levels such as AST and ALT are a good indicator of liver damage [18]. The elevated serum levels of AST and ALT in the present study indicate a reduction and/or loss of functional integrity of hepatocyte membranes. These laboratory findings may therefore be useful to predict the prognosis of patients with acute PQ poisoning. For example, they could help clinicians make timely and accurate assessments of patient condition in acute stage of poisoning. However, some groups reported no significant differences in ALT and AST between survival and death groups after PQ poisoning [9, 19–21]. These disparate results may be due to differences in patient age and the ingested dose.

Renal function-related indicators such as K^+^ and Cl^−^ were closely associated with renal damage in mice and humans. Changes in electrolytes also had a certain value for predicting outcomes of patients with PQ poisoning. ROC curve analyses confirmed that K^+^ and Cl^−^ are valuable prognostic indicators. Differences in K^+^ and Cl^−^ concentrations were significantly associated with mortality in patients with PQ poisoning (*P* < 0.05). Patients who died showed significant reductions in Cl^−^ and K^+^ concentrations compared to those in the survival group. Compared with the PQ200 and PQ100 groups, Cl^−^ concentration was reduced in the PQ360 group (lethal dose). The Cl^−^ concentration trends in the PQ mouse model and patients were consistent, but there was no significant difference in Cl^−^ concentration in mice. We speculate that the reason for this inconsistency is the difference in measurement time. Animal serum electrolyte levels were measured 24 h after PQ ingestion, and K^+^ significantly increased in the PQ360 group. Conversely, levels were significantly decreased in patients in the death group. It is generally considered that hypokalemia caused by PQ poisoning might be due to four possible mechanisms. (1) Loss of K^+^ from the kidneys: PQ induces the formation of free oxygen radicals, which contributes to renal tubular necrosis and affects K^+^ re-absorption [22]. (2) Loss of K^+^ and phosphorus via the gastrointestinal tract: blood electrolyte levels were measured for all patients within 1 h of admission, but gastrointestinal tract impairment is a chronic process, so this factor might not be the main cause of hypokalemia. (3) Transfer of extracellular K^+^ into cells: increased secretion of catecholamines and glucocorticoids and enhanced activity of sodium-potassium pump in response to oxidative stress caused by PQ poisoning will promote K^+^ entry into cells and lead to decrease serum K^+^ levels [23]. (4) Diuretic use to promote PQ excretion after poisoning may lead to significant renal K^+^ excretion. The increase of K^+^ in mice is likely due to decreased food and water intake of mice after ingesting PQ, which affects kidney function and prevents K^+^ excretion. Acute renal failure is a well-recognized complication of PQ poisoning as the compound is primarily metabolized by the kidneys, causing renal injuries such as proximal tubular necrosis [24, 25] and Fanconi syndrome [26]. The specific mechanism underlying this effect is unclear, but protecting the kidneys in the early stage of PQ poisoning and promoting toxin excretion can reduce organ damage, improve patient prognosis, and increase the survival rate.

This study explored and compared prognostic indicators between an animal model and patients. We found that the combination of age, urinary PQ concentrations volume of PQ ingested, K^+^, Cl^−^, BUN, ALT, AST, amylase, and NLR can accurately predict the outcome of patients with PQ poisoning. Our results show that different ingested PQ volumes had a statistically significant impact on patient mortality (*P* < 0.05), which was confirmed by ROC curve analysis. Larger ingested PQ volumes will lead to higher plasma PQ concentrations and poorer prognostic outcomes. We also found that patients with higher urinary PQ concentrations had poorer prognostic outcomes, which is consistent with a report by Deng et al. [27]. BUN level had a statistically significant impact on the mortality of patients with PQ poisoning (*P* < 0.05) in our results; however, others stated that although BUN was increased in the death group, there were no significant differences between survival and death groups [22, 23]. Song and colleagues found the mean lethal PQ dose ingested by patients was 60 ml (50-100 ml), which was lower than that in our study (128.00 ± 61.97 ml). In addition, the mean age of the death group patients in our study was older than that in the study by Zhang et al.

As a sensitive infection indicator, NLR is considered as a product of inflammation stress response that can predict patient outcome [23]. PQ can affect inflammation in vitro [28] and cause neutrophilia and lymphocytopenia in acute poisoning patients [29]. In this study, patients with higher NLRs had poorer outcomes. Routine blood routine tests revealed that NLR was significantly associated with mortality of patients with PQ poisoning (*P* < 0.05). However, ROC curve analysis showed that NLR was not a powerful prognostic indicator compared with other measures. When the NLR cut-off value was 15.00, sensitivity was 70.00% and specificity was 94.70% for prediction mortality. This is inconsistent with previous research [29, 30]. Analysis on the results of pancreatic function examinations for patients with PQ poisoning revealed that amylase level was significantly associated with mortality (*P* < 0.05), in that patients with higher amylase concentrations had poorer outcomes. This is consistent with the results of two recent studies [31, 32]. A related investigation suggested that amylase was strongly associated with the severities of nephrotoxicity, hepatotoxicity, and pancreas injury induced by PQ [33]. However, one group suggested that amylase levels in PQ poisoning patients were not significantly different between the death and survival groups [21], so further verification is needed.

## 5. Conclusion

In summary, the combination of age; urinary PQ concentration and ingested volume; NLR; liver function indicators ALT and AST; kidney function indicator BUN; and serum levels of K^+^, Cl^−^, and amylase can more accurately predict the prognosis of patients with PQ poisoning. Our results show that the indicators in mice are not identical to those of humans. There were differences in most biochemical parameters, except for liver function and some renal function indicators. Overall, the C57BL/6J mouse strain is a valuable animal model for research on liver and renal function in subjects with PQ poisoning.

## Figures and Tables

**Table 1:** Comparison of test results between the survival group and the death group.

Characteristics	Survival group (*n* = 19)	Death group (*n* = 10)	*P* value
Sex			0.095
Male	12 (63.16%)	3 (30%)	
Female	7 (36.84%)	7 (70%)	
Age (yr)			0.006^∗∗^
<20	1 (5.26%)	0	
20~40	10 (52.63%)	2 (20%)	
>40	8 (42.11%)	8 (80%)	
Ingested volume (ml)	25.26 ± 17.28	128.00 ± 61.97	*P* ≤ 0.001^∗∗^
Urinary PQ concentration (ug·ml^−1^)	16.1 ± 10.80	85.33 ± 32.97	0.007^∗∗^
MAP (mm Hg)	99.58 ± 10.20	103.97 ± 9.01	0.263
RR (min^−1^)	19.05 ± 3.60	21.50 ± 6.02	0.170
WBC (10^9·L^−1^)	10.39 ± 4.44	13.52 ± 4.81	0.090
NLR	9.46 ± 5.05	15.04 ± 8.14	0.030^∗^
K^+^ (mmol·ml^−1^)	3.85 ± 0.30	3.45 ± 0.50	0.023^∗^
Cl^−^ (mmol·ml^−1^)	105.58 ± 2.73	101.30 ± 9.20	0.021^∗^
AST (U·L^−1^)	22.05 ± 8.49	107.50 ± 201.41	0.019^∗^
ALT (U·L^−1^)	23.74 ± 15.03	137.30 ± 233.20	0.033^∗^
BUN (mmol·ml^−1^)	5.29 ± 3.05	9.69 ± 7.96	0.033^∗^
Cr (umol·L^−1^)	63.57 ± 21.01	126.11 ± 150.13	0.872
PT (s)	11.45 ± 2.82	14.12 ± 6.56	0.335
APTT (s)	32.57 ± 20.24	33.00 ± 20.65	0.836
D-D (mg·L^−1^)	0.59 ± 0.86	3.70 ± 9.69	0.136
Amylase (U·L^−1^)	66.00 ± 29.32	211.33 ± 141.42	0.009^∗∗^

Abbreviations: ALT: alanine aminotransferase; APTT: activated partial thromboplastin time; AST: aspartate aminotransferase; BUN: blood urea nitrogen; Cl^−^: serum chlorine; Cr: creatinine; D-D: D dimer; K^+^: serum potassium; MAP: mean arterial pressure; NLR: neutrophil-to-lymphocyte ratio; PQ: paraquat; PT: prothrombin time; RR: respiratory rate; WBC: white blood cell.

**Table 2:** ROC curve analysis.

Variable	Area under ROC curve	95% CI	Cutoff point	Sensitivity (%)	Specificity (%)	Youden index (%)
Age	0.779	0.597-0.961	>48.00	90	68.40	58.40
Ingested volume	0.953	0.875-1.00	>75.00	80	100	80
Urinary PQ concentration	0.883	0.749-1.00	>82.50	80	86.70	66.70
AST	0.832	0.677-0.986	>24.50	80	78.95	58.95
ALT	0.845	0.700-0.990	>44.50	60	94.74	54.74
BUN	0.797	0.619-0.976	>7.17	60	94.70	54.70
K^+^	0.761	0.553-0.968	<3.72	80	68.40	48.40
Cl^−^	0.763	0.558-0.969	<103.00	60	89.50	49.50
Amylase	0.889	0.726-1.00	>136.00	66.67	100	66.67
NLR	0.737	0.504-0.970	>15.00	70	94.70	64.70

Abbreviations: ALT: alanine aminotransferase; AST: aspartate aminotransferase; BUN: blood urea nitrogen; CI: confidence interval; Cl^−^: serum chlorine; K^+^: serum potassium; NLR: neutrophil-to-lymphocyte ratio; PQ: paraquat; ROC, receiver operating characteristic.

## Data Availability

The [DATA TYPE] data used to support the findings of this study are included within the article.

## Supplementary Materials

Supplementary Materials Supplementary Figure 1: flowchart of experimental. Supplementary Figure 2: effect of PQ on mouse body weight over 20 consecutive days. Statistical analyses were performed using two-way ANOVA, and individual group differences were measured using Tukey's multiple comparisons tests. ^∗^*P* < 0.05, ^∗∗^*P* < 0.01, ^∗∗∗^*P* < 0.001 versus control; ^#^*P* < 0.05, ^##^*P* < 0.01, ^###^*P* < 0.001 versus sham. Supplementary Figure 3: the effect of PQ on other indicators in mice. A: Mg^2+^; B: P; C: ALP; D: SA; E: TBIL; F: db; G: IBIL; H: A/G; I: TBA; G: BUN; K: CRE; L: *β*2M/BMG.
