# Corrigendum to “Comparison of Biochemical Parameters between Mouse Model and Human after Paraquat Poisoning”

**DOI:** 10.1155/bmri/9827387

**Published:** 2025-07-14

J. Yu, L. Zhang, X. Li, et al., “Comparison of Biochemical Parameters between Mouse Model and Human after Paraquat Poisoning,” *BioMed Research International* 2022 (2022): 1254824, https://doi.org/10.1155/2022/1254824.

In the above article, published on January 28, 2022, there was an error in Figure 1. In the keys for panels “a” and “b”, the group name “PQ 300” should be “PQ 360.” The corrected figure is shown below, and is listed as Figure 1.

We apologize for this error.

## Figures and Tables

**Figure 1:**
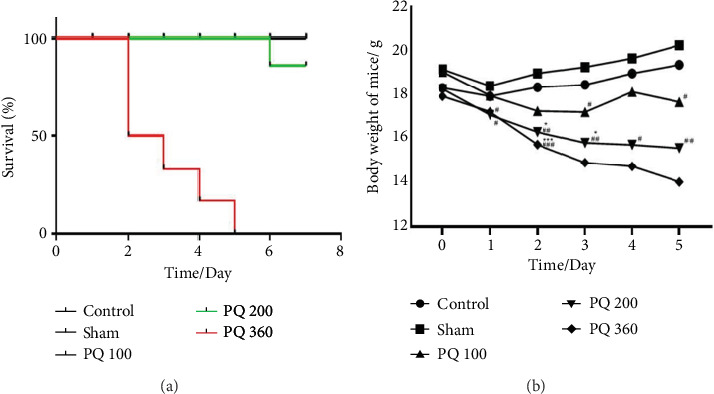
Effect of PQ on mouse body weight and survival rate. (a) Survival time was recorded over 7 days. (b) Body weight change was monitored for 5 days. PQ intoxication caused marked weight loss. Statistical analyses were performed using two-way ANOVA, and individual group differences were measured using Tukey's multiple comparisons tests. ⁣^∗^*p* < 0.05, ⁣^∗∗^*p* < 0.01, and ⁣^∗∗∗^*p* < 0.001 versus control; #*p* < 0.05, ##*p* < 0.01, and ###*p* < 0.001 versus sham.

