# Supplementary material for: Comparison of Biochemical Parameters between Mouse Model and Human after Paraquat Poisoning
Source: Biomed Res Int. 2022 Jan 28;2022:1254824. doi: 10.1155/2022/1254824 (PMC8816545; doi:10.1155/2022/1254824)
Supplement: Supplementary Materials — Supplementary Figure 1: flowchart of experimental. Supplementary Figure 2: effect of PQ on mouse body weight over 20 consecutive days. Statistical analyses were performed using two-way ANOVA, and individual group differences were measured using Tukey's multiple comparisons tests. ∗P < 0.05, ∗∗P < 0.01, ∗∗∗P < 0.001 versus control; #P < 0.05, ##P < 0.01, ###P < 0.001 versus sham. Supplementary Figure 3: the effect of PQ on other indicators in mice. A: Mg2+; B: P; C: ALP; D: SA; E: TBIL; F: db; G: IBIL; H: A/G; I: TBA; G: BUN; K: CRE; L: β2M/BMG. [file 1254824.f1.zip › Supplementary Figure 2.pdf]

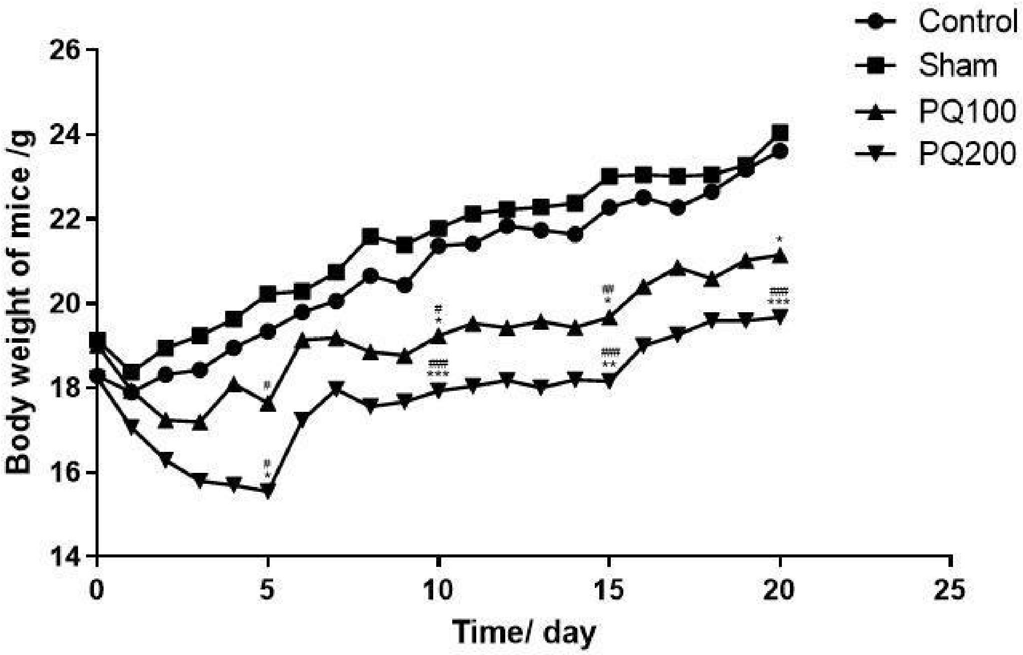

Supplementary Figure 2. Effect of PQ on mouse body weight over 20 consecutive days. Statistical analyses were performed using two-way ANOVA, and individual group differences were measured using Tukey's multiple comparisons tests. *$P<0.05$, **$P<0.01$, ***$P<0.001$ versus control; $^{\#}P<0.05$, $^{\#\#}P<0.01$, $^{\#\#\#}P<0.001$ versus sham.
