# Supplementary material for: Comparison of Biochemical Parameters between Mouse Model and Human after Paraquat Poisoning
Source: Biomed Res Int. 2022 Jan 28;2022:1254824. doi: 10.1155/2022/1254824 (PMC8816545; doi:10.1155/2022/1254824)
Supplement: Supplementary Materials — Supplementary Figure 1: flowchart of experimental. Supplementary Figure 2: effect of PQ on mouse body weight over 20 consecutive days. Statistical analyses were performed using two-way ANOVA, and individual group differences were measured using Tukey's multiple comparisons tests. ∗P < 0.05, ∗∗P < 0.01, ∗∗∗P < 0.001 versus control; #P < 0.05, ##P < 0.01, ###P < 0.001 versus sham. Supplementary Figure 3: the effect of PQ on other indicators in mice. A: Mg2+; B: P; C: ALP; D: SA; E: TBIL; F: db; G: IBIL; H: A/G; I: TBA; G: BUN; K: CRE; L: β2M/BMG. [file 1254824.f1.zip › Supplementary Figure 3.pdf]

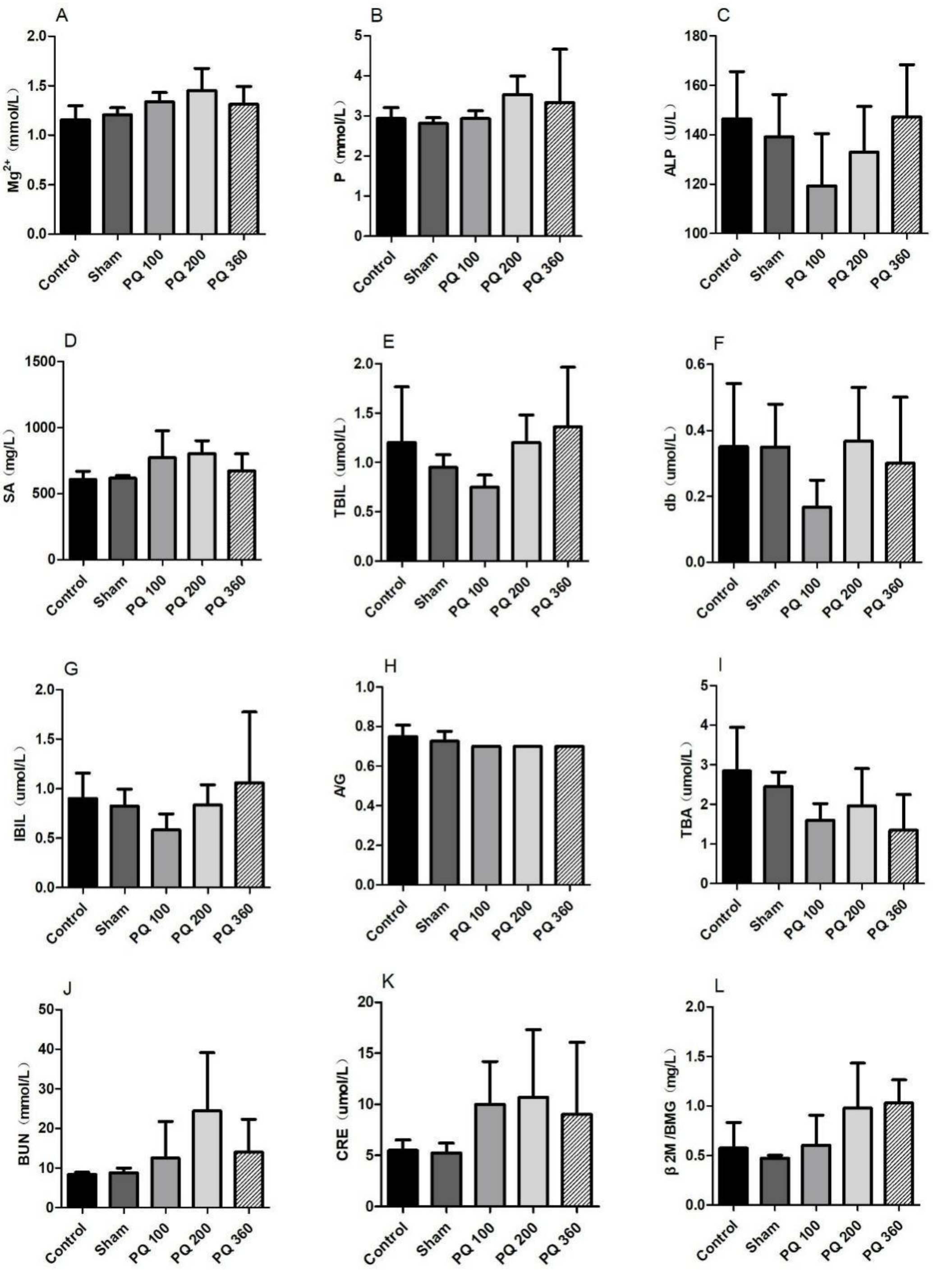

Supplementary Figure 3. The effect of PQ on other indicators in mice. A: Mg^{2+}; B: P; C: ALP; D: SA; E: TBIL; F: db; G: IBIL; H: A/G; I: TBA; G: BUN; K: CRE; L: β2M/BMG.
